# Combination of Piezoelectric and Triboelectric Devices for Robotic Self-Powered Sensors

**DOI:** 10.3390/mi12070813

**Published:** 2021-07-12

**Authors:** Zhicheng Han, Pengchen Jiao, Zhiyuan Zhu

**Affiliations:** 1 Institute of Port, Coastal and Offshore Engineering, Ocean College, Zhejiang University, Hangzhou 316021, China; hzc244097644@email.swu.edu.cn; 2 College of Electronic and Information Engineering, Southwest University, Chongqing 400700, China

**Keywords:** piezoelectric devices, triboelectric devices, robotics, self-powered sensors

## Abstract

Sensors are an important part of the organization required for robots to perceive the external environment. Self-powered sensors can be used to implement energy-saving strategies in robots and reduce their power consumption, owing to their low-power consumption characteristics. The triboelectric nanogenerator (TENG) and piezoelectric transducer (PE) are important implementations of self-powered sensors. Hybrid sensors combine the advantages of the PE and TENG to achieve higher sensitivity, wider measurement range, and better output characteristics. This paper summarizes the principles and research status of pressure sensors, displacement sensors, and three-dimensional (3D) acceleration sensors based on the self-powered TENG, PE, and hybrid sensors. Additionally, the basic working principles of the PE and TENG are introduced, and the challenges and problems in the development of PE, TENG, and hybrid sensors in the robotics field are discussed with regard to the principles of the self-powered pressure sensors, displacement sensors, and 3D acceleration sensors applied to robots.

## 1. Introduction

With the development of science and technology, intelligent robots have been developed through the fusion of multiple technologies, including machine vision technology, communication technology, sensor technology, and even biotechnology. Intelligent robots have been introduced into various fields, in addition to the traditional industrial manufacturing fields [1], such as underwater detection, medical treatment, service, and other fields [2,3,4,5]. The working environment of intelligent robots is also becoming increasingly complex. To improve the intelligence of robots, human control interventions must be reduced when the environment changes, the robot’s perception of the working environment and its own state must be improved, and corresponding feedback actions and posture adjustment must be carried out according to the situation. The robots’ detection of the environment mainly relies on vision and various sensor types. Machine vision allows robots to approximately model the environment, but the perception accuracy is low. For example, when a robot is grabbing objects, it cannot directly understand the grasping force through vision. Although machine learning is used to replace touch sensors for object perception [6,7,8], machine vision is far from sufficient for achieving precise control and coping with complex and changing environments.

In recent years, research on soft robots, and particularly research on soft touch sensors applied to robots [9,10,11], has intensified. Compared with traditional touch sensors, soft touch sensors have higher sensitivity and can perceive more subtle tactile information from the external environment. Moreover, soft sensors have a lower Young’s modulus and can better adapt to the environment through deformation [12]. In particular, the interaction between robots and humans has become safer and more comfortable [1,13,14]. Robots need increasingly more sensors [14], which requires smaller sensors, higher integration, and lower power consumption. Because self-powered sensors can use their own energy to provide information, they have lower power consumption and only need two wires for signal transmission, and can even can realize signal transmission with only one wire. Therefore, self-powered sensors have been widely investigated [15,16,17].

Piezoelectric and triboelectric energy are important implementation methods for self-powered sensors. The piezoelectric transducer (PE) uses piezoelectric materials. When the piezoelectric materials deform, they are internally polarized. Two types of piezoelectric materials are typically used, namely crystal materials and ceramic materials. Crystal materials have limitations such as high production cost, sensitivity to moisture, and difficulty in obtaining consistent orientations. However, polycrystalline ceramic materials are easy to process [17], and have relatively low manufacturing cost, higher sensitivity than crystals [18], and a wide range of applications. In recent years, structures such as nanofibers [19], nanowires [20], nanoparticles [21], nanocubes [22], and nanorods [23] have been used in piezoelectric material structures. Among them, nanofibers and nanowires can effectively convert the strain caused by low-strength mechanical external forces into electrical energy [19]. This piezoelectric transducer with nanostructures is known as the piezoelectric nanogenerator (PENG). Piezoelectric materials combined with self-sensing technology can be used as a sensor while acting as an actuator [24,25]. The main advantages of self-sensing are the embeddability of measurement technology, low cost and lack of a need for additional sensors [24]. Through piezoelectric actuator self-sensing technology, precise positioning applications such as micromanipulation and microassembly can be realized [25,26,27,28,29,30].

The main source of triboelectricity is the principle of triboelectric nanogenerators (TENGs). The triboelectric nanogenerator invented by Wang et al. in 2012 uses the triboelectric effect and electrostatic induction effect to convert mechanical energy into electrical energy [31]. The TENG structure is simple, easy to manufacture, and has low cost. Owing to these characteristics, it is more convenient to use a TENG to convert wind energy [32], water wave energy [33], and the mechanical energy of human movement into electrical energy [34]. As a self-powered sensor, the TENG has good output characteristics, as has been demonstrated by studies considering pressure sensors, displacement sensors, and inertial sensors. Additionally, because the TENG can flexibly select materials, and the materials can be soft materials [35], it can be effectively applied to soft robots [36].

**Figure 1:**
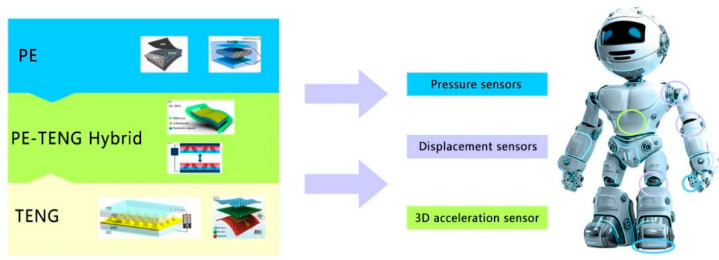
Illustration of PE (reproduced with permission from references [40,41]), TENG (reproduced with permission from references [42,43]), and PE–TENG hybrid (reproduced with permission from references [44,45]).

Owing to their particular structure and material characteristics, the PE sensor and TENG have their particular advantages and disadvantages in different application scenarios. Sensors made by combining the PE and TENG have higher sensitivity, wider measurement range and better output characteristics [37]. Owing to their outstanding characteristics, the PENG and TENG have been extensively investigated in recent years, and it has been found that these sensors have good development potential [38]. The artificial intelligence design model provides a new method for designing, predicting and optimizing the structure and materials of PENG and TENG [39]. The sensors implemented in robots mainly include pressure sensors, displacement sensors, and three-dimensional (3D) acceleration sensors. The motion perception and environment perception of a robot are inseparable from these three types of sensors. Based on research pertaining to PE, TENG, and PE–TENG hybrid (PTENG) sensors, this paper summarizes the principles used by these three sensors to realize pressure sensing, displacement sensing, and three-dimensional (3D) acceleration sensing, and their corresponding applications in the field of robotics (Figure 1). In the first part, the principles of the PE and TENG are briefly introduced. The next few sections introduce the research results obtained for PE, TENG, and hybrid self-generation sensors in the field of pressure sensors, displacement sensors, and spatial acceleration sensors over the last year. Additionally, the sensors’ potential applications in the field of robotics are introduced. Finally, the opportunities, challenges, and future expectations for PE sensors, TENG sensors, and PE–TENG hybrid sensors in the practical application of robot perception are introduced.

## 2. Principle and Process of PE and TENG

This chapter briefly explains the principles of PE and TENG and the research status of materials and the manufacturing processes in recent years. Owing to their characteristics, PE and TENG are generally used to measure dynamic data.

### 2.1. Piezoelectric Effect and Piezoelectric Materials of PE

**Figure 2:**
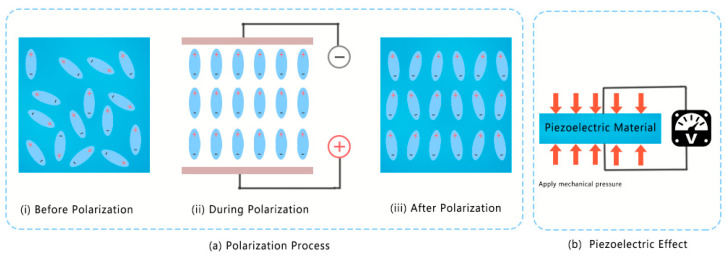
(**a**) Polarization process; (**b**) piezoelectric effect.

Piezoelectric materials are polarized by a strong electric field in a specific direction at high temperature, such that the randomly oriented electric dipoles in the material can be arranged in an orderly manner. Furthermore, they induce the piezoelectric effect (Figure 2a). When external pressure is applied to the piezoelectric material, it causes the polarization of the piezoelectric material (Figure 2b). When the external electric field acts on the piezoelectric material, it will cause the deformation of the piezoelectric material. Therefore, piezoelectric materials can realize self-sensing based on these two reversible effects. In this way, the information of displacement, force and state in the micro-robot can be estimated by piezoelectric devices [24,25].

The piezoelectric properties of piezoelectric materials involve the interaction of electrical and mechanical behavior. The piezoelectric effect is expressed as follows [46]:

(1) $$S_{i}=c_{ij}\sigma_{j}+d_{ki}E_{k}$$

(2) $$D_{m}=d_{mj}\sigma_{j}+\varepsilon_{mk}E_{k}$$

where *D* and *E* are the electric displacement vector and electric field vector; *S* and *σ* are the strain vector and stress vector; *c* is the flexibility matrix; *d* is the piezoelectric material constant; *ε* is the dielectric constant; *m*, *k* = 1,2, 3, and *i*, *j* = 1, 2, 3, 4, 5, 6 represent different directions in the Cartesian coordinate system.

Originally, natural materials, such as quartz and Rochelle salt, were used as piezoelectric materials to develop underwater acoustic piezoelectric devices that can detect submarines. With the development of piezoelectric materials, materials such as piezoelectric ceramics, piezoelectric polymers, and ferroelectrics have gradually replaced the expensive quartz. Piezoelectric ceramics and ferroelectric solid solutions are fabricated by synthesizing mixtures of different oxides, carbonates, and metal salts, which can be customized according to different actual usage scenarios. Among them, the most widely used material is the ceramic-based lead zirconium titanate (PZT) (Pb[Zr_(x)_Ti_(1−x)_O_3_]), which has superior piezoelectric properties and is widely used in sensors, drivers, and electronic components. However, the amount of lead in lead-based piezoelectric materials generally exceeds 60 wt%. Hence, during the production and recycling process of PZT, the release of lead, mainly in the form of lead oxide or lead zirconate titanate, is very large and causes substantial environmental pollution [47,48]. Therefore, mainly lead-free materials have been considered in recent piezoelectric materials research [49,50,51].

Common lead-free piezoelectric ceramics with perovskite structure are mainly based on barium titanate (BaTiO_3_, BT), bismuth sodium titanate (Bi_0.5_Na_0.5_TiO_3_, BNT), potassium sodium niobate (K_0.5_Na_0.5_NbO_3_, KNN), and so on. Among them, the BT-based perovskite structure has excellent piezoelectric, ferroelectric, and dielectric properties, and the preparation process is compatible with the traditional lead-based ceramic process. Hence, this has become the most widely investigated structure with the best development potential in recent years [52,53]. Current research mainly considers three aspects for improving the piezoelectric performance of lead-free piezoelectric ceramics. On one hand, the piezoelectric performance is improved through different preparation methods and preparation processes, while on the other hand, the piezoelectric properties of the material are improved through the doping modification of the material composition [50,54]. In addition, the output characteristics can be improved by designing a new structure [55].

### 2.2. Principles and Process of TENG

The TENG mainly uses frictional electrification and electrostatic induction. Because different substances have different abilities for gaining and losing electrons, when two substances are in contact, the material with a relatively strong ability to gain electrons will absorb electrons from the other material. After the two materials are separated, a potential difference will exist between the two materials owing to the difference in the number of electrons and different charges [31,56,57]. The working mode can be divided into the contact separation mode, lateral sliding mode, single-electrode mode, and independent layer mode. The contact separation mode is simple, and relatively easier for understanding and analyzing the system. An overview of the TENG’s working principle through the working cycle of the contact separation mode is provided below.

Generally, the two materials of the contact layer have a relatively high dielectric constant, and a certain gap exists between the gain and loss of the charge ability of the two materials. In other words, certain materials such as wool, melamine, and so on, can be easily charged, while other materials, such as Teflon, PVC, and so on, can easily lose charge. Hence, a metal material is attached to the exterior contact layer [2,58].

**Figure 3:**
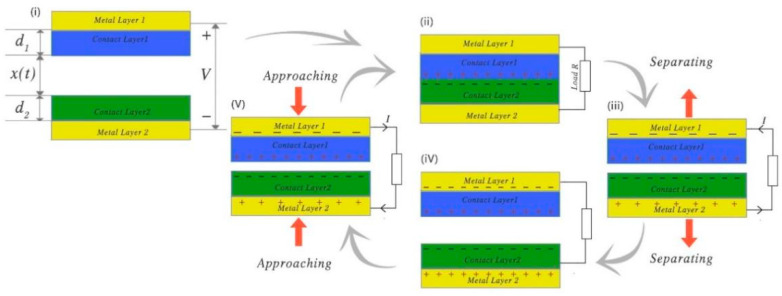
Power generation principle of contact separation TENG type: (**i**) The initial state of the device; (**ii**) two contact layers are in contact; (**ii**)–(**iv**) The process of two contact layers separating; (**iv**)–(**ii**) the process of two contact layers approaching.

Figure 3i shows that, when the two contact layers are not in contact, both materials are charged and are in a state of charge balance. As shown in Figure 3ii, when two contact layers are in contact, charge transfer occurs between the two contact layers owing to the triboelectric effect. At this time, the charges are on the same plane and there is no induced electric potential to the exterior. When the contact layer is separated, an electric potential is generated between the two contact layers. Owing to the electrostatic induction effect, the metal layer at the exterior of the contact layer will generate a corresponding induced electric potential. By connecting the two metal layers, the charge is transferred by the drive of the electric potential until the two metal layers reach a state of charge balance. When the two contact layers are close again, owing to the electrostatic induction, the potential between the two metal layers changes in the opposite direction, and a reverse current is generated until the electric charges of the metal layers reach equilibrium again. The two metal layers serve as the output electrodes of this TENG structure. In this working mode, an alternating current is formed between the two electrodes in each cycle.

Studies have systematically established theoretical models for the above-mentioned situation and clarified the TENG’s output characteristics. In the TENG system, the important dynamic control parameter is the distance x between the two plates, and the important output parameters are the induced potential V between the two electrodes and the amount of charge Q transferred between the two electrodes.

As shown in Figure 3, the dielectric constants of the two contact surfaces are $\varepsilon_{1}$ and $\varepsilon_{2}$, and the thicknesses are $d_{1}$ and $d_{2}$, respectively. The change of the distance x between the two contact surfaces is driven by the external force. The actual distance between the two contact surfaces that changes with time t is denoted as x(t). Under ideal conditions, without considering the edge effect, the electrons on the metal electrode are evenly distributed. Under this condition, the output voltage $V_{oc}$ and current $I_{sc}$ of the two metal electrodes can be deduced according to electrodynamics [58]:

(3) $$V_{oc}=\frac{\sigma x\left(t\right)}{\varepsilon_{0}}$$

(4) $$I_{sc}=\frac{dQ_{sc}}{dt}=\frac{S\sigma \cdot \left(\frac{d_{1}}{\varepsilon_{1}}+\frac{d_{2}}{\varepsilon_{2}}\right)}{{\left(\frac{d_{1}}{\varepsilon_{1}}+\frac{d_{2}}{\varepsilon_{2}}+x\left(t\right)\right)}^{2}}\cdot \frac{dx\left(t\right)}{dt}$$

Therefore, $V_{oc}$ is related to the distance *x*. $Q_{sc}$ is transferred charge. When the gap *x* reaches the minimum, $V_{oc}$ also reaches the minimum. When the gap *x* increases, $V_{oc}$ increases until it reaches the maximum value. Moreover, it can be seen that the open circuit voltage $V_{oc}$, charge transfer amount σ, and open circuit current $I_{sc}$ are all proportional to the charge density σ. Therefore, increasing the maximum charge density *σ* of the contact surface is very important for improving the output capability of the TENG. The short-circuit current $I_{sc}$ is related to the speed at which the distance between the contact surfaces changes.

From the above analysis, it can be seen that there are several approaches toward improving the TENG’s output capacity. One approach is to increase the contact area. The structure of the contact surface is realized by a certain microstructure, and studies have used this method to improve the TENG’s output characteristics [1,59]. Increasing the triboelectric effect can effectively increase the charge density of the material and thereby improve the TENG’s output capability. To this end, microstructures can be made on the contact surface and functional materials can be used [42,60,61]. According to the output characteristics of TENG, the current change between the two electrodes can be used to dynamically detect the speed of the change in the distance between the two contact surfaces [62,63].

We have summarized the working modes of TENG, PENG, and PE–TENG hybrid in the actual application. A general comparison between each principle, for each application situation, is shown in Table 1.

## 3. Pressure Sensors

### 3.1. PE Pressure Sensors

The PE pressure sensor is based on the pressure effect. Under the application of external mechanical pressure, a piezoelectric material will be polarized, which eventually leads to the electron transfer of the piezoelectric semiconductor device and results in current change. Therefore, the dynamic change of the current can reflect the dynamic change of the piezoelectric material caused by an external force [84]. Based on this principle, many piezoelectric diodes and piezoelectric transistors that can be used as pressure sensors have been investigated [85,86]. Among them, the piezoelectric field effect tube investigated by Kang et al. [87] has linear characteristics when the external force is close to a Nanonewton.

**Figure 4:**
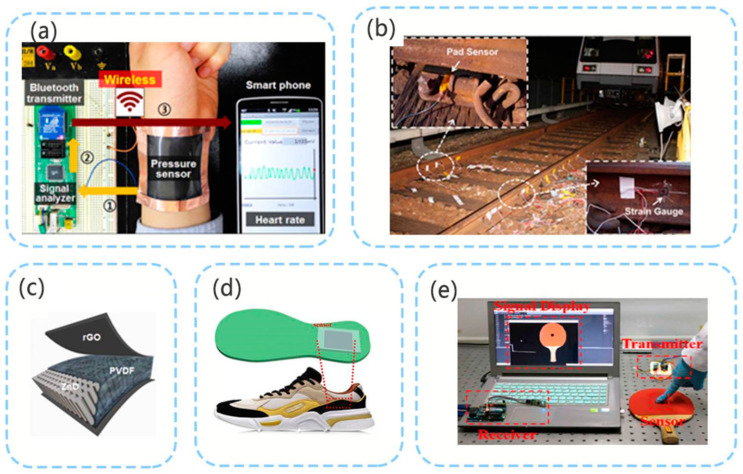
(**a**) PE pressure sensor for detecting heart rate (reproduced with permission from reference [40]); (**b**) PE pressure sensor for load measurement on a track (reproduced with permission from reference [64]); (**c**) structure of PE pressure sensor [58] (reproduced with permission from reference [40]); (**d**) PE pressure sensor on sole for detecting different types of human motion (reproduced with permission from reference [65]); (**e**) PE pressure sensor installed on racket to obtain hit data (reproduced with permission from reference [66]).

As shown in Figure 4a, in 2016, Shin et al. [40] developed a sensor for detecting the heart rate based on a ZnO nanoneedle/polyvinylidene fluoride hybrid film. The minimum detection pressure of this sensor can reach 4 Pa. In this study, a ZnO nanostructure with a hexagonal protrusion of two and high aspect ratio was used as shown in Figure 4c to reduce the elastic modulus of the hybrid film. In 2018, Zhang et al. [64] used a polyvinylidene fluoride-based multi-layer sensing device and installed it under a train track for load measurement on the track. Both the experimental test and the actual dynamic test revealed that good performance characterized by good linearity was achieved. Owing to its low production cost and easy installation, this sensor can be used as a vehicle for weighing in motion, and can also detect the surface wear of running vehicles in real time (Figure 4b). Many studies have investigated the detection of human movement and contact through PE sensors. Yang et al. [65] installed a pressure sensor on a sole (Figure 4d) to detect different types of human motion and changes in joint motion based on the signal. In 2019, the PZT/PVDF piezoelectric sensor designed by Tian et al. [66] was installed onto a table tennis racket. This sensor can detect the location where the athlete hit the ball with the racket and the strength of the shot, and the obtained data can be used to evaluate the athlete’s movement (Figure 4e). Many studies on detecting human movement and contact through PE pressure sensors have also been conducted [41,88]. High-sensitivity PE sensors can even detect nearby drops and small vibrations [89].

### 3.2. TENG Pressure Sensors

The TENG pressure sensor mainly uses the contact separation mode of the TENG, and has been extensively investigated because it has a simple structure and is easy to implement.

As can be seen from the theoretical model of the contact separation mode of the TENG, the output characteristics of the TENG exhibit a linear relationship with the distance between the contact surfaces. An air gap exists when the contact surfaces of the TENG are in contact. When external pressure is applied, the gap decreases. When the external pressure is removed, the gap becomes maximum; that is, the open circuit voltage Voc reaches the maximum. Studies have shown that the friction nanogenerator can measure pressure [90].

Using the spring model and Hooke’s law, the relationship of the distance *x* between two contact surfaces and the pressure *p* can be obtained as follows:

(5) $$p=\frac{k\cdot \Delta x}{S}=\frac{k\cdot \left(x_{0}-x\right)}{S}$$

where *k* is the elastic coefficient of the material. By combining Equation (3), the following relationship can be obtained:

(6) $$\frac{V_{oc,0}-V_{oc}}{V_{oc,0}}=\frac{x_{0}-x}{x_{0}}=\frac{S}{k\cdot x_{0}}\cdot p$$

From Equation (6), it can be seen that the open circuit voltage of the TENG has a linear relationship with the pressure, and this relationship can be used to make static pressure observations. The previously derived short-circuit current can be observed, and the dynamic rate of change of the distance between the two contact surfaces can be derived from the short-circuit current. Using this feature, the external force loading rate can also be dynamically detected.

Generally, the TENG is optimized in two ways to improve the sensing output characteristics of the piezoelectric sensor. The first approach is to increase the area of the contact surface so as to improve the triboelectric effect. A larger contact surface area can accumulate more charges. The contact surface is micro-processed; for example, the surface has a pyramidal structure [42,91,92,93]. Because the cost of micro-processing is relatively high, the contact surface is also polished with sandpaper to increase the roughness of the contact surface and contact area [60]. Moreover, the use of electrospinning technology can greatly increase the TENG output. Guo et al. [94] proposed an all-fiber hybrid piezoelectric-enhanced triboelectric nanogenerator with a power density of 310 µW cm^−2^, which was fabricated using electrospinning silk fibroin and poly(vinylidene fluoride) (PVDF) nanofibers in conductive fabrics. Electrospinning also provides a good solution for using the TENG in human body induction and energy harvesting applications [95].

The second method is to use a porous structure or a material with low Young’s modulus as the contact surface material. The use of a porous structure can reduce the material density, improve the deformability of the sensor [96,97], and increase the initial distance between the two contact surfaces. In addition to the above-mentioned methods, recent studies have used the external-charge pumping method to increase the charge density of the friction layer and thereby increase the TENG output [98]. In the study by Liu et al. [99], both the external charge excitation and self-charge excitation in a TENG system were realized using voltage-multiplying circuits. The effective charge output density of the TENG system reached 1.25 m·cm^−2^.

**Figure 5:**
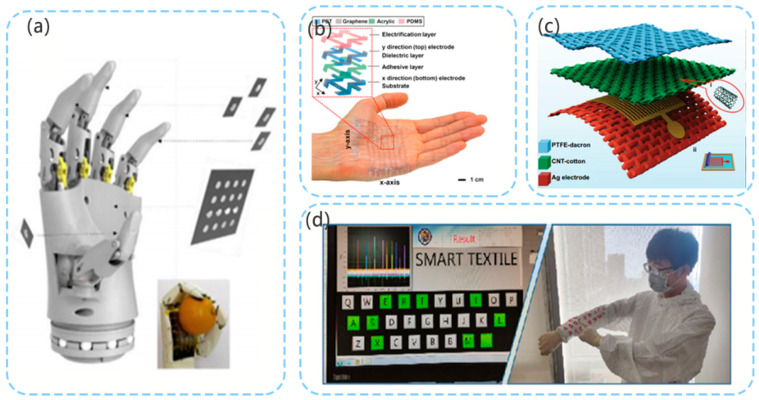
(**a**) Electronic skin of robot (reproduced with permission from reference [73]); (**b**) flexible TENG pressure sensor arranged in 8 × 8 array (reproduced with permission from reference [74]); (**c**) structure of PE pressure sensor (reproduced with permission from reference [43]); (**d**) self-powered wearable keyboard (reproduced with permission from reference [43]).

Both the TENG pressure sensors and PENG sensors have high sensitivity. In the study by Zhang et al. [73], a capacitive sensor was combined with a TENG sensor for use in the electronic skin of a robot (Figure 5a). Capacitive pressure sensors have good linear characteristics when measuring high pressures. The high sensitivity of the TENG sensor was used to detect the external force in the pressure range of <10 kPa, while the capacitive sensor was used to detect the external force of 10−120 kPa. This combination greatly expands the measurement range of the pressure sensor and improves its sensitivity. In this study, liquid metal was used as an electrode, and silicone rubber was used as the dielectric material and packaging material, which endowed the electronic skin with good ductility. Lee et al. [74] developed a stretchable TENG touch sensor. This sensor uses atomic thin graphene as the electrode, polyethylene terephthalate as the substrate, and polydimethylsiloxane as the electrification layer. Stretchability is achieved through the implementation of a grid structure. In this study, an 8 × 8 array was used, and the contact position was measured by considering the pressure (Figure 5b). In 2021, Wang’s team used silver-plated fabric, carbon nanotubes, and polytetrafluoroethylene to make a retractable TENG sensor (Figure 5c) [43]. The average instantaneous current density of the sensor reached 170 µA·m^−2^. In this study, the sensor was also used to develop a self-powered wearable keyboard (Figure 5d).

### 3.3. PE–TENG Hybrid Pressure Sensor

**Figure 6:**
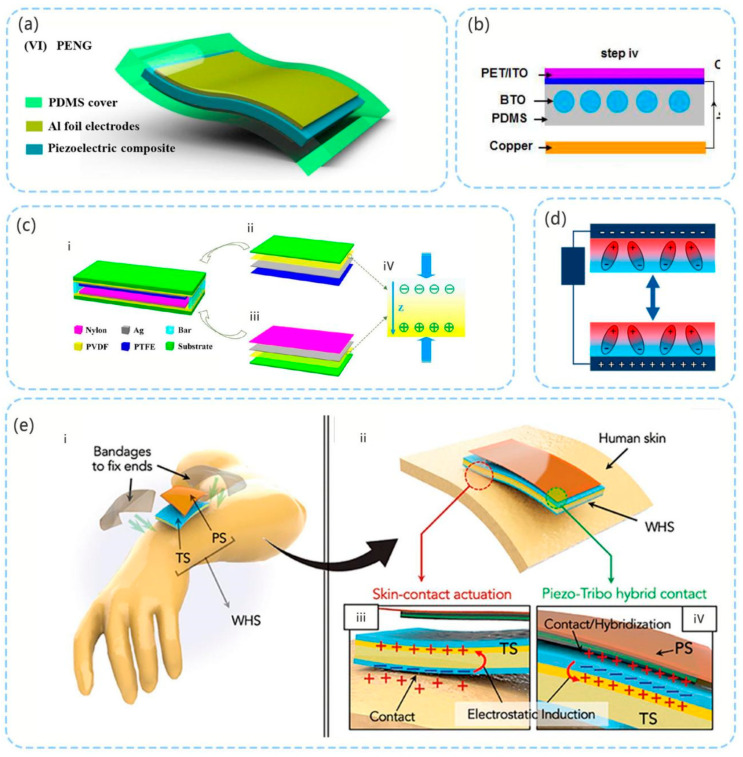
(**a**) Flexible pressure sensor developed using regenerated C/BT aerogel paper-based PDMS nanocomposites (reproduced with permission from reference [44]); (**b**) composite film comprising ferroelectric BTO NPs in PDMS polymer matrix (reproduced with permission from reference [80]); (**c**) schematic structure of double-piezoelectric-layer-enhanced TENG (reproduced with permission from reference [81]): (**i**) schematic structure of the device, (**ii**) detailed composition layers of top section and (**iii**) bottom section, (**iv**) the polarization direction of PVDF; (**d**) schematic diagram of using two piezoelectric layers as friction layers (reproduced with permission from reference [45]); (**e**) representation of working principle of three-electrode hybrid sensor (reproduced with permission from reference [82]): (**i**) representation (exploded view) of the applicability of the device onto the human skin, (**ii**) representation of the working principle of the device, (**iii**) triboelectric coupling with the human skin, (**iv**) PE-TENG hybrid contact.

Hybrid pressure sensors are generally realized by superimposing the PE and TENG in the direction of the mechanical force. In the study by Shi et al. [44], flexible high-performance pressure sensors were developed using regenerated cellulose/BaTiO_3_ (C/BT) aerogel paper-based polydimethylsiloxane (PDMS) nanocomposites (Figure 6a). With the introduction of the single-electrode mode TENG, the maximum output power could reach 85 μW. Suo et al. [80] developed a composite film comprising ferroelectric barium titanate (BTO) nanoparticles (NPs) in a PDMS polymer matrix [80] (Figure 6b), and demonstrated through experiments that the output performance of the hybrid sensor is better compared with that of the PE and TENG. Notably, the TENG uses a contact separation mode.

To improve performance, a double piezoelectric layer is used to increase the polarization effect. In the study by Xia et al. [81], the conductive Ag layer was placed between the PVDF film and the friction contact layer. The conductive Ag layer serves as the output electrode of the device [81] (Figure 6c). A nylon membrane and PTFE membrane are used as the upper and lower friction contact layer, respectively. The maximum output power density of the device achieved 185 μW·cm^−2^ with a match load of 9 MΩ. In the study by Linards et al. [45], two ferroelectric PVDF/BaTiO_3_ nanocomposite films were used as the PE part and also as the TENG contact layer [45]. The conductive electrode is located at the exterior of the device (Figure 6d). A dual-capacitor model of the device’s operating mode was proposed. In the experiment, the open circuit voltage of the device reached 2.7 kV from 5 cm^2^.

Mariello et al. [82] combined the PE and TENG to make a three-electrode hybrid sensor. The PE part is based on transparent lead-free inorganic aluminum nitride (AIN) film sputtering deposited on a flexible substrate. The TENG part comprises an ultra-soft patch of DMS and Ecoflex mixture, which is encapsulated in a parylene friction film. This sensor has three types of electrical signals that trigger release: piezoelectric, skin-contact actuation, and piezotribo hybrid contact. The sensitivity that can be triggered is higher, and the detection range of small and irregular movements increases. The TENG uses two modes, namely, the contact separation and single-electrode modes (Figure 6e).

Table 2 summarizes the output characteristics and sensor characteristics of the above-mentioned sensors. As can be seen from the summary, the sensor sensitivity can improve the detection accuracy of the sensor very well. After the sensitivity of the sensor meets the actual requirements, the strength of the signal energy output can be improved, which is helpful to improve accuracy, noise immunity and signal recognition [100].

## 4. Displacement Sensors for Robotics

As an important unit of robot perception, displacement sensors are used in many scenarios. Both the robot’s motion feedback and perception of the external environment require the participation of displacement sensors. The displacement sensor mainly detects linear displacement and rotational displacement, and obtains dynamic acceleration information in the displacement direction. In recent years, piezoelectric displacement sensors have mainly been used for positioning detection at the nm/µm level. The measurement range is small, and most importantly, the low-frequency characteristics are poor [67,68]. Therefore, this sensor is mainly used to measure vibrations, sound waves [101], and so on. This chapter mainly summarizes sensors related to robot motion displacement; therefore, the above-mentioned sensor types are not discussed in detail.

The TENG displacement sensors generally use the TENG’s sliding mode. The displacement sensor realizes the sliding type of the TENG, and there are two main structural realization methods. The first method is to divide the two contact layers into a certain regular arrangement. When the contact layers are close, the metal layer outside of the contact layer generates an induced electric potential owing to electrostatic induction. When the two contact layers are separated, a reverse potential is generated. In the second method, the sensing metal outside of the contact layer and the contact surface are divided into a certain regular shape or matrix arrangement. When the contact surface moves owing to the change of the electric field, the induced metal close to the electric field undergoes charge transfer owing to electrostatic induction.

**Figure 7:**
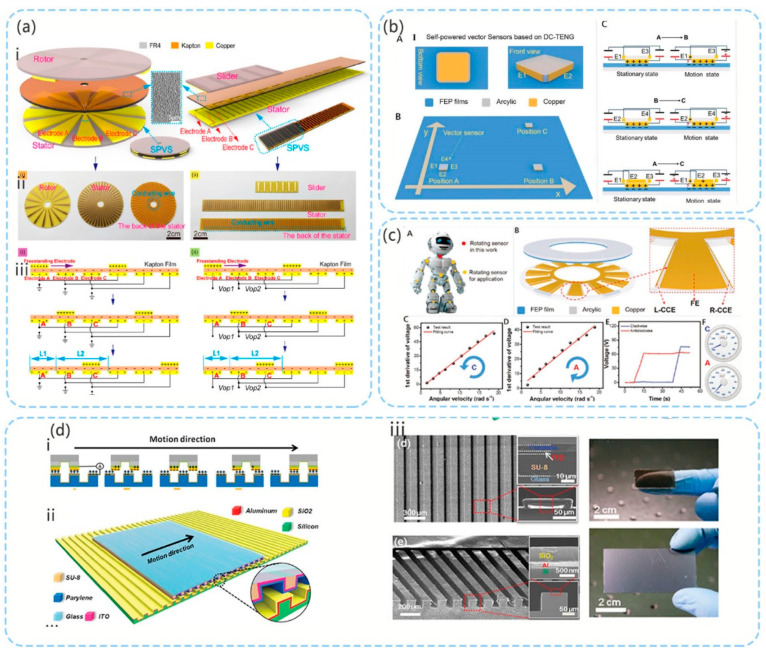
(**a**) TENG displacement sensor (reproduced with permission from reference [75]); (**b**) TENG plane displacement sensor (reproduced with permission from reference [76]); (**c**) TENG rotary displacement sensor (reproduced with permission from reference [76]); (**d**) TENG high-precision displacement sensor (reproduced with permission from reference [102]).

In the study by Li et al. [75], the second above-mentioned method was used for design, and an accuracy of 8°/5 mm was achieved. As shown in Figure 7a, when the upper contact layer slides, the contact layer causes charge transfer between the upper and lower contact layers owing to the triboelectric effect. When the metals on the outer side of the upper contact layer and lower contact layer overlap owing to electrostatic induction, the metal in the overlapping area is positively charged, while the metal in the non-overlapping area is negatively charged. Through different circuit connection methods, different potential difference signals are obtained, and the direction of movement, distance, and acceleration can be calculated from these signals.

As shown in Figure 7b, in the study by Yin et al. [76] based on the TENG, the electrostatic breakdown effect was used to construct a DC sensor. The sensor mainly comprised four charge collection electrodes (CCE: E1, E2, E3 and E4) and a common friction electrode (FE). The structure is shown in Figure 7b. The CCE layer is placed around the sensor and close to the triboelectric plane to cause air breakdown. The FE slides on the fluorinated ethylene propylene (FEP) plane. A 3.6 nF capacitor exists between the FE and CCE layers. On one hand, this capacitor is used to store electrons generated when the FE and FEP are rubbed. On the other hand, the motion parameters can be evaluated by considering the voltage of the capacitor. When the FE slides on the FEP plane, the FEP plane and FE layer will generate charges owing to the triboelectric effect. Because the electric field between the surface of the FEP and CCE is sufficient for breaking down air, the electrons transferred through breakdown can be stored in the capacitor. If the FEP plane and FE layer continue to rub, the transfer of electrons will continue. By assessing the voltage of the capacitors connected to different CCEs, the movement direction can be determined. In this study, the first derivative of voltage had a good linear relationship with speed, while the second derivative of voltage had a strong linear relationship with the acceleration. This sensor has high sensitivity and, because the output is a DC signal, it is free of interference from electromagnetic signals in the environment. As shown in Figure 7c, the study also verified that this sensor can measure the rotational displacement and linearly detect the direction, angular velocity, and acceleration information of the rotational movement within a certain range. The measurement results also have relatively good linear characteristics.

Because the accuracy requirements of robot sensors are continuously increasing, in the study by Zhou et al. [102], the first above-mentioned structure was implemented in the design and a high-precision displacement sensor was developed (Figure 7d). A displacement resolution of 173 nm was achieved within a working distance of tens of millimeters, and the linearity error was 0.02%.

The upper and lower contact surfaces are made of xylene and silicon dioxide materials, and a certain material is used as the substrate. Additionally, micro grating is formed by micro processing. Xylene is more triboelectrically negative compared with silica. The upper and lower gratings are charged after sliding contact. Owing to electrostatic induction, the metal layer of the takeaway fabric generates an induced electrical potential, and the movement information can be calculated by detecting the open circuit voltage and short circuit current.

**Figure 8:**
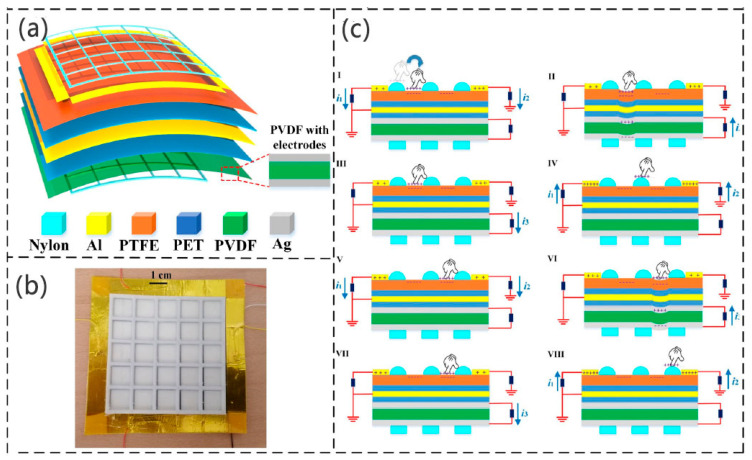
Device configuration and operation mechanism of hybrid sensor (reproduced with permission from reference [103]): (**a**) enlarged 3D view of device showing the detailed layer-by-layer structure; (**b**) photograph of device placed on table; (**c**) operation mechanism of device for position and force sensing.

In 2021, Liu et al. developed a displacement pressure sensor using the PE–TENG hybrid, which can obtain displacement information and contact information [103] (Figure 8). The TENG uses the single-electrode mode. The sensor structure comprises an upper nylon mesh layer, four aluminum (Al) electrodes, a polytetrafluoroethylene (PTFE) friction layer, a polyethylene terephthalate (PET) insulation layer, an aluminum shielding layer, another PET insulating layer, a PVDF piezoelectric layer with two silver (Ag) electrodes, and a lower nylon mesh support layer (Figure 8a). The friction layer has four output electrodes, which serve as the edge detection of the grid. When a finger touches the friction layer, a friction charge is generated and the touch position is detected by comparing the detection output ratio. In the initial condition, the finger is far away from the PTFE surface, and the entire system is in an electrostatic equilibrium state. The fingers (bare or wearing nitrile gloves) will be positively charged after contacting the PTFE friction layer, and the PTFE surface will be negatively charged at the same time. The electrons will flow to the four friction electrodes according to the potential difference, and the flow of electrons closer to the contact position will increase. The contact position can be detected by the output ratio of the four rubbing electrodes. Moreover, the pressure of the finger’s touch is applied to the PVDF layer, which results in electric dipolarization inside the piezoelectric layer. The electric charge that drives the electrodes of the piezoelectric layer will flow. When the PVDF layer recovers from deformation, the piezoelectric charges will flow in the opposite direction. By detecting the output charge difference and piezoelectric current of the friction electrode, the position information and pressure information of single-point contact and multi-point contact can be obtained.

## 5. Space Acceleration Sensor for Robot

The main application of the displacement sensor is to obtain displacement, velocity, and acceleration information in a specific direction or at a specific angle. In actual robot applications, spatial acceleration sensors are also indispensable and essential for determining the motion state of the robot. Moreover, a space acceleration sensor can be used to obtain collision and vibration information.

### 5.1. PE 3D Acceleration Sensor

**Figure 9:**
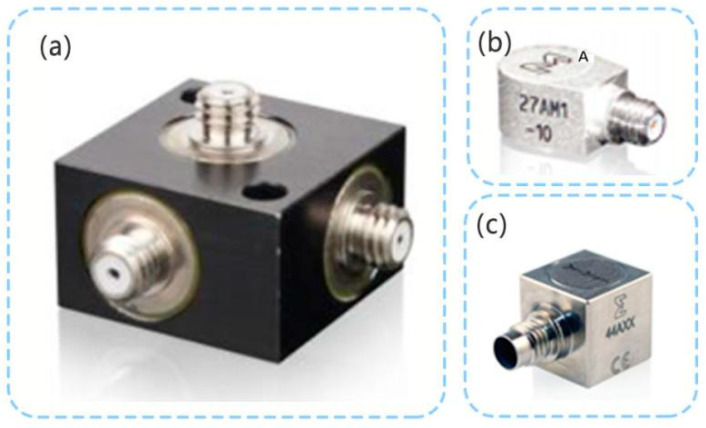
(**a**) ENDEVCO PE 3D acceleration sensor; (**b**) ENDEVCO single-axis IEPE (weight: 1 g; size: 8 × 6 × 5 mm^3^); (**c**) ENDEVCO 3D IEPE.

The PE 3D acceleration sensor is different to the TENG 3D acceleration sensor. The previously developed PE3D accelerometer has a long history of research, design, manufacturing, and application [70,104], and is considered to be mature. Figure 9a shows a PE 3D accelerometer developed by ENDEVCO. This PE accelerometer does not contain electronic circuit components and can work at higher temperatures. For example, some piezoelectric materials allow the PE accelerometer to work at 650 °C [71,72,105], or even at 900 °C [106].

Presently, there are piezoelectric accelerometers (IEPE) with built-in circuits. The IEPE integrates signal amplification, filtering, and other circuits, which are widely used in the field of dynamic vibration measurement. In this sensor, the PE accelerometer is used as the mechanical part of the IEPE, and the electronic circuit is used as the electrical part [69,107,108,109]. As shown in Figure 9b, in ENDEVCO products, the single-axis IEPE can have the minimum size of 8 × 6 × 5 mm^3^ with a weight of only 1 g. Figure 9c shows the 3D IEPE of ENDEVCO.

In EPE sensors, PE does not provide the sensor’s back-end circuit processing energy source, but only serves as a capacitive signal source with high impedance. When the IEPE is working, an external power supply is required to provide energy for the sensor’s internal signal processing circuit. In recent years, some studies have used the PE for self-generated 3D acceleration, and only a few studies have used the PE as the power source for a 3D acceleration sensor [110]. Hence, the processing of the PE acceleration sensor’s output signal should be further investigated [111,112].

### 5.2. TENG and Hybrid 3D Acceleration Sensor

**Figure 10:**
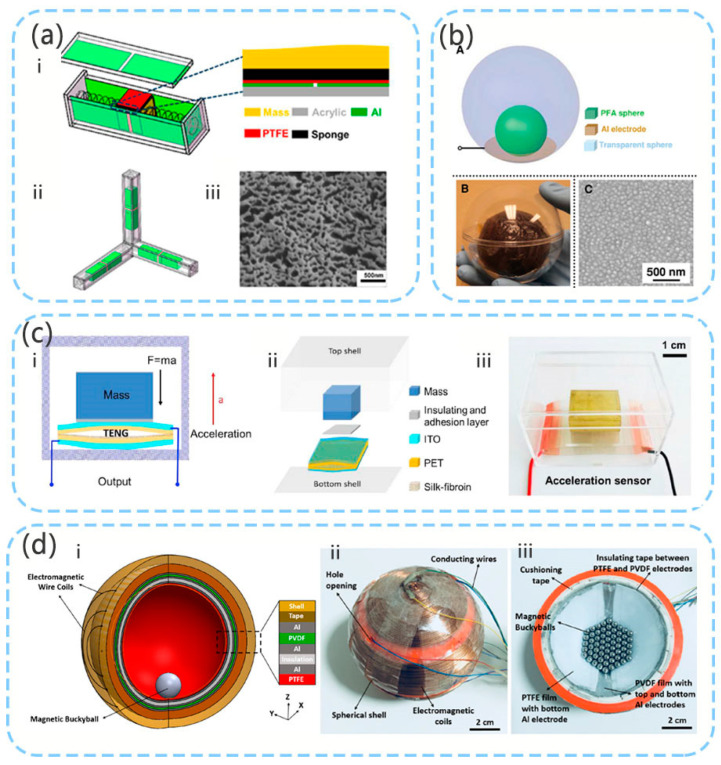
(**a**) TENG 3D acceleration sensor (reproduced with permission from reference [77]); (**b**) spherical TENG 3D acceleration sensor (reproduced with permission from reference [113]); (**c**) self-powered and high sensitivity TENG acceleration sensor (reproduced with permission from reference [78]); (**d**) self-powered 3D activity inertial sensor using hybrid sensing mechanisms (reproduced with permission from reference [83]).

The structure of the TENG friction sensor comprises a mass, an elastic damping structure, and a power generating body. The 3D acceleration sensor has been investigated by Peng et al. [77] (Figure 10a). The three-dimensional sensor calculates the spatial acceleration through measurement in three mutually perpendicular directions. An interior spring is used as an elastic damping structure. In TENG’s horizontal sliding mode, when the mass block slides, the contact surface on the mass block slides relative to the surface of the container. This structure has better output characteristics under low frequency operation. Sensors with this structure generally have better output effects and robustness. However, because the structure requires support, the overall volume is relatively large, which is not conducive to the use of small structures.

In the study by Liu et al. [78], the contact mode of TENG was used, and an elastic material was used to form an arched structure for supporting the mass (Figure 10c). This study proposed the V-Q-a theoretical model of a self-powered high-sensitivity acceleration sensor based on the TENG. This sensor can achieve acceleration in the range of 1–11m/s^2^ with a sensitivity of up to 20.4 V/(m/s^2^) and power density of up to 371.8 mW/m^2^. Based on this structure, Liu et al. [79] subsequently used silk-fibroin as the triboelectric layer to validate the model, and the sensitivity of the sensor reached 19.07 V/(m/s^2^).

The study by Liu et al. [113] proposed a novel structure without support (Figure 10b). This structure also uses TENG’s contact separation mode. However, the contact area of the triboelectric material of TENG is relatively small. The study by Koh et al. [83] considered a spherical structure, but this gyro ball uses TENG, piezoelectric, and electromagnetic hybrid energy collection (Figure 10d). In addition to measuring the acceleration of the three spatial axes, the sensor can also be used as a rotation acceleration sensor for measuring the roll and pitch.

## 6. Concluding Remarks

This paper summarizes the pressure sensors, motion sensors, and 3D acceleration sensors that have been used in robots in recent years, and briefly describes the working principles, structures, and characteristics of these sensors. As a research hotspot in recent years, self-powered sensors have developed rapidly. Among them, the PE and TENG are important self-powered sensors and have been extensively investigated. Research on PE sensors started relatively early, and relatively mature technologies exist with regard to the materials, structure, and signal processing of self-powered sensors. Particularly, acceleration sensors have been widely used. In recent years, PENG research has greatly improved the sensitivity of PE sensors, particularly with regard to pressure sensors. Owing to the structural characteristics of its material, the PE is mainly used in high-precision displacement sensors and small-range displacement detection applications, and has certain frequency requirements. Owing to their structural characteristics, TENG sensors have great development potential as self-powered pressure sensors, self-powered displacement sensors, and self-powered acceleration sensors. Among them, the TENG self-powered displacement sensor has unique advantages. The TENG displacement sensor has a large range, even in high-precision displacement measurement, and good output characteristics. The PE–TENG hybrid sensor combines the advantages of the PE and TENG, and has higher sensitivity, wider measurement range, and better output characteristics. It is important that different structural designs of the PE–TENG hybrid can detect more information regarding various robot movements.

Self-powered sensors also have unique advantages in low-power electronic devices. It is very important for low power electronic devices to improve energy conversion efficiency through the optimization of self-powered sensors. In recent years, there have been many achievements in the research on improving the interface of self-powered sensors [114,115] for enhancing the energy conversion efficiency [116,117,118]. Because TENG sensors will be used in practical or even commercial applications, many aspects should be investigated in future work. The PE–TENG hybrid acceleration sensor still requires extensive research with regard to miniaturization. The size of the hybrid sensor is still relatively large, and reducing the volume of the self-powered sensor will inevitably reduce the sensor’s output. Maintaining higher sensor output with smaller sensor volume is also a topic that should be investigated. In sensor research, self-powered hybrid sensors use the advantages of different sensors to greatly improve their measurement range and output characteristics. Hence, self-powered hybrid sensors are also a promising field for future sensor research.

## Figures and Tables

**Table 1:** Application comparison of PE, TENG and PE–TENG hybrid.

Technologies	Pressure Sensors	Displacement Sensors	Space Acceleration Sensor
PE (Refs. [24,25,40,64,65,66,67,68,69,70,71,72])	Good output characteristics under low pressure; Self-sensing	Nm-level high-frequency displacement measurement; Small measuring range; Self-sensing	Mature technology and application; Less affected by ambient temperature; Unstable output under low measurement frequency
TENG (Refs. [43,73,74,75,76,77,78,79])	Mainly use contact separation mode or single-electrode mode of TENG; Good output characteristics under low pressure	Mainly use sliding model of TENG; Good measuring range, high measuring accuracy	Mainly use contact separation mode or single-electrode mode of TENG; Good low-frequency output characteristics; Unable to measure high-frequency vibration
PE–TENG Hybrid (Refs. [44,45,80,81,82,83])	Better output characteristics, more detection content (e.g., measuring the bending direction)	Mainly use contact separation mode or single-electrode mode of TENG; Good output characteristics and low measurement accuracy; Mainly used in human-computer interaction button detection	Good low-frequency output characteristics; Unable to measure high-frequency vibration

**Table 2:** Output and sensitivity of pressure sensors.

Technologies	Energy Output	Sensitivity	Range	Ref.
PE	-	0.8 V/kPa	-	[41]
	-	>4 Pa	-	[40]
	Power density of 1.22mW·m^−^^2^ with load resistance of 70 M	Output voltages 0.1, 0.28, 0.45 V with various angles (around 60°, 90°,120°)	-	[65]
	V_oc_: 2.51 V, I_sc_: 78.43 nA	6.38 mV/N	-	[66]
TENG	-	1.04 V/kPa (<10 kPa) Strain sensitivity 1.23 (10~120 kPa)	0~120 kPa	[73]
	V_oc_: 15.1 V I_sc_: 4.7 uA Power density of 36 μW·m^−^^2^ with 30 kPa of pressure	0.274 V/kPa (10.6 kPa~101.7 kPa)	1.3 kPa~101.7 kPa	[74]
	Power density of 3.8 mW∙m^−^^2^, Average current density of 170 μA·m^−^^2^ with load resistance of 1 GΩ	-	-	[43]
PE–TENG Hybrid	V_oc_: 2.7 kV Power density of 1.85 mW·m^−^^2^ with load resistance of 9 MΩ. Output voltage: 48 V Output power: 85 μW	-	-	[44]
	V_oc_: 2.7 kV Power density of 1.157 W·m^−^^2^ with load resistance of 1 MΩ. Charge density (6.55 nC·cm^−^^2^).	-	-	[45]
